# Visual acuity disorder and its relationship with work and extra work factors in workers of a marketplace in a Colombian municipality, 2017-2018

**DOI:** 10.47626/1679-4435-2023-1138

**Published:** 2024-11-14

**Authors:** María Osley Garzón Duque, Santiago Jaramillo Guzmán, Manuela Jiménez Cifuentes, Fabio Leon Rodríguez Ospina

**Affiliations:** 1 Medical School, Universidad CES, Medellín, Antioquia, Colombia; 2 National Faculty of Public Health, Universidad de Antioquia, Medellín, Antioquia, Colombia

**Keywords:** occupational diseases, occupational health, informal sector, visual acuity, myopia, degenerative, enfermedades profesionales, salud laboral, sector informal, agudeza visual, miopía degenerativa

## Abstract

**Introduction:**

The loss of visual acuity represents a public health problem of interest that also affects the living and working conditions of informal workers; however, this is still a poorly explored topic.

**Objectives:**

To identify the prevalence of loss of visual acuity in workers in the marketplace of a Colombian municipality.

**Methods:**

This is a cross-sectional study with a primary source of information from a census of 194 workers from a marketplace, to whom an assisted survey was applied and an occupational medical evaluation was carried out. Univariate, bivariate and multivariate analyzes were performed.

**Results:**

Study population was predominantly female, 60.8% had a partner, more than half were between 14 and 44 years old, 78.2% of them worked > 8 hours a day, 9.2% had a diagnosis of diabetes, 37.7% of arterial hypertension, and 15.2% had a visual acuity disorder. Greater loss of visual acuity in older workers (prevalence ratio =21.07, confidence interval 5,15-86.17), in men (prevalence ratio = 1.65, confidence interval 1.21-2.25), in those who perceived that they had loss of visual acuity (prevalence ratio =2.08, confidence interval 1.45-2.99) and blurred vision (prevalence ratio =2.74, confidence interval 1.39-5.39) and in those who had arterial hypertension (prevalence ratio =2.28, confidence interval 1.25-4.17).

**Conclusions:**

Actions are necessary from the municipal health and labor authorities and from national programs, so as to allow workers and their families to take responsibility for individual activities to promote visual health and to participate in collective actions to advance in the modification of the conditions that make it difficult for them to have good visual health.

## Abstract (es)

**Introducción:**

La pérdida de la agudeza visual representa un problema de interés en salud pública que también afecta las condiciones de vida y la labor de trabajadores informales; sin embrago, este aun es un tema escasamente explorado.

**Objetivos:**

Identificar la prevalencia de pérdida de la agudeza visual en trabajadores de la plaza de mercado de un municipio colombiano.

**Métodos:**

Se trata de un estudio transversal con fuente primaria de información, a un censo de 194 trabajadores de una plaza de mercado, a quienes se les aplicó encuesta asistida y se les realizó evaluación médica ocupacional. Fueron realizados análisis univariados, bivariados y multivariados.

**Resultados:**

Población predominantemente femenina, 60,8% tenía pareja, más de la mitad tenía entre 14 y 44 años, y 78,2% de ellos laboraba > 8 horas al día. El 9,2% tenía diagnóstico de diabetes, el 37,7% de hipertensión arterial, y un 15,2% presentó trastorno de agudeza visual. Mayor pérdida de agudeza visual en trabajadores mayores (razón de prevalencia = 21,07, índice de confiabilidad 5,15-86,17), en los hombres (razón de prevalencia = 1,65, índice de confiabilidad 1,21-2,25), en quienes percibían que tenían pérdida de agudeza visual (razón de prevalencia = 2,08, índice de confiabilidad 1,45-2,99) y visión borrosa (razón de prevalencia = 2,74, índice de confiabilidad 1,39-5,39), y en los que tenían hipertensión arterial (razón de prevalencia = 2,28, índice de confiabilidad 1,25-4,17).

**Conclusiones:**

Son necesarias acciones de las autoridades de salud y trabajo municipal y de programas a nivel nacional, que les permitan a los trabajadores y a sus familias hacerse responsables de actividades individuales de promoción de la salud visual y participar de acciones colectivas para avanzar en la modificación de las condiciones que les dificultan tener buena salud visual.

## INTRODUCTION

According to data from the World Health Organization (WHO), at least 2.2 billion people have visual impairment, and the prevalence of visual impairment is four times higher in lowand medium-income regions.^1^ In countries such as Ecuador, Venezuela, and Colombia, prevalence is estimated between 10% and 13%,^2^ percentages in line with the reported by the Colombian National Institute for the Blind (Instituto Nacional de Ciegos de Colombia, INCI), totaling 1,100,000 people with visual impairment (of which 80% are visually-disabled and 20% are blind).^3^ The main causes of visual impairment and blindness are uncorrected reflective errors and cataract, whose onset could have been prevented or corrected in 50% of the cases.^1^

Visual impairment is associated with mental health impairment, major cognitive impairment, and high risk of falls and other injuries. It is also strongly associated with sociodemographic factors and access to health services,^4^ causing a strong impact on quality of life, particularly among adults, because visual impairment reduces their participation in the labor market and their productivity rates.^1^

The WHO global report on vision, adopted by the 73rd World Health Assembly in 2020, intends to convert integrated and person-centered ophthalmic care into the care model of choice, so as to ensure its universal provision in order to reduce the burden of eye conditions and visual impairment, in addition to favoring the accomplishment of the Sustainable Development Goals (SDGs).^1^

In Colombia, the 2016-2022 National Program of Comprehensive Visual Health Care fosters actions that provide the population with tools to maintain good visual health and incorporate interventions that allow for delivering timely and high-quality care, with special emphasis on groups in situations of inequality.^5^ Among these groups there are informal workers, as defined by the Colombian National Administrative Department of Statistics (Departamento Administrativo Nacional de Estadística, DANE), including groups of subsistence workers who do not rely on social security and experience worse health and hygiene conditions than those of formal workers.^6^ In Colombia, for July 2019, 47.5% of the working population had informal jobs,^6^ a percentage that increased with the emergence of the pandemic. In the case of the municipality where the study was conducted, the rate of informality was 87.48% in 2019.^7^ For the workers of the marketplace where the present study was conducted, there is still scarce information on their health and working conditions, a situation that makes it difficult both for workers and for municipal managers and health authorities to guide health promotion and disease prevention actions.^8^

Although the relationship between visual health and working conditions, habits, and lifestyles has not been well defined yet, visual protection has been acknowledged as one of the preventable limitations to intervene in the informal work sector, according to the “Strategy of healthy work environment with emphasis on the informal sector”, proposed by the Colombian Ministry of Health in 2018,^9^ since limitations caused by eye conditions and visual impairment can be prevented or resolved, and its progression can be prevented in a cost-effective and feasible manner, by means of lenses, rehabilitation, surgery, among others.^10^

Despite the foregoing, and although there are international and national initiatives aiming to improve visual health and contribute to better social inclusion of individuals affected by vision loss,^11^ some barriers coexist, such as lack of information that makes it possible to characterize the informal workers’ population the impact of vision loss on their health, which is necessary for the planning of public and health policies that enables to address visual impairment. Due to the reasons previously described, the present study aims to identify the prevalence of loss of visual acuity and its relationship with sociodemographic, occupational, and health characteristics of marketplace workers in a municipality of Southwestern Antioquia, Colombia, to facilitate the prioritization of actions with and for workers, focusing on their actual conditions and perceived needs.

## METHODS

### DESIGN

A cross-sectional study with analytical approach was conducted using primary information sources, through the administration of an assisted survey and workers’ medical/occupational assessment, after signature of informed consent and/or assent.

### POPULATION

The survey was administered to 194 marketplace workers in a municipality of Southwestern Antioquia, Colombia, in December 2017. The study and all its activities were arranged with the board of directors of the marketplace, as well as with workers, the Municipal Department of Health, and the School of Medicine of Universidad CES.

### INCLUSION AND EXCLUSION CRITERIA

The study included workers aged 14 years or older, with at least one year of work experience, who were familiar of the study activities, and who agreed to participate. According to the established exclusion criteria (not answering ≥ 50% of the survey, not signing the informed consent and/or assent, being under the effect of psychoactive substances), no participant was lost. The study was conducted in accordance with the ethical standards of the Declaration of Helsinki and Resolution 8340 of 1993 of Colombia; furthermore, it was classified as a minimum risk study and was approved by the Institutional Research Ethics Committee of Universidad CES, under protocol 123.

### VARIABLES

Dependent - loss of visual acuity (Yes/No), assessed through the Snellen chart, using the following ranges: 20/20, 20/25, 20/30, 20/40, 20/50, 20/70, 20/100, 20/200. This chart was re-categorized according to the WHO scale and the International Classification of Diseases, 10th revision (ICD-10) into: normal/mild loss of visual acuity (20/20 - 20/70); moderate loss of visual acuity (worse than 20/70 - 20/200); and severe loss of visual acuity (worse than 20/200).

Independent - sociodemographic characteristics; age, sex, marital status, age in years, affiliation to the health system, type of affiliation, and worker’s socioeconomic status.

Working conditions - working hours per day, type of product sold, and type of lighting in the workplace.

Comorbidities -personal or familial history of diabetes, personal or familial history of hypertension, personal or familial history of cancer, personal history of drug use.

Visual symptoms in the last year and last month- self-report of: reduced visual acuity, changes in eye color, blurred vision, double vision, seeing holes or black spots, eye dryness, and headache.

Medical assessment was performed by two professor physicians and by fourth and fifth medical students of Universidad CES. Data from the survey were obtained by professors and medical students of Universidad CES, students of Health Information System Management of Universidad de Antioquia, and a group of assistants from the Health Department and of the municipal hospital, at workers’ workplaces and the second floor of the marketplace.

### CONTROL OF BIAS

Selection bias was controlled by performing a workers’ census for the study, and information bias was controlled by raising awareness of the workers’ population, the marketplace manager, hospital personnel, and staff of the municipal department of health, which were standardized with the researchers and field work assistants. A pilot test was conducted with three workers located in areas surrounding the marketplace, in order to make adjustments to the instrument and to enhance the data collection process.

### ANALYSIS

#### Univariate analysis

Frequency and percent distributions were calculated for qualitative variables. Descriptive statistics including measures of central tendency, position, and dispersion were calculated for quantitative variables, which were also assessed through the Kolmogorov-Smirnov (K-S) test to define the type of bivariate tests to be performed. Age and daily working hours were re-categorized for bivariate analysis.

#### Bivariate analysis

Bivariate analysis was performed with chi-square test to establish the association between loss of visual acuity and independent qualitative variables under study. Median differences (Mann-Whitney *U*) were used to relate aged and loss of visual acuity. Prevalence ratios (PR) were calculated to determine the strength of association between prevalence of loss of visual acuity and independent variables.

#### Multivariate analysis

Binary logistic regression analysis with explanatory purposes was performed, including variables that met the Hosmer-Lemeshow (HL) goodness-of-fit criteria (p < 0.25). Analysis of information was performed on, Epidat 3.1 and 4.2, and SPSS 21 software licensed to Universidad CES. All statistical tests were performed with a 95% confidence level and 5% margin of error.

## RESULTS

### SOCIODEMOGRAPHIC AND WORKING CONDITIONS, COMORBIDITIES, AND SYMPTOMS REPORTED BY WORKERS

It was observed that 56.7% of this population was women, 47.4% was older than 45 years, and 60.8% had a partner at the time of data collection. With regard to their monthly income, 72.4% reported that it was below the current legal minimum monthly salary, although 78.2% worked more than 9 hours daily. In relation to their working environmental conditions, 95.9% of workers considered that their workplace was illuminated, mainly with lamps (79.9%) (Table 1).

**Table 1:** Frequency and percentage distribution of workers’ sociodemographic and working conditions, comorbidities, and symptoms (n = 194)

	n	%
Sociodemographic variables		
Sex		
Men	84	43.3
Women	110	56.7
Marital status		
With a partner	118	60.8
Without a partner	75	38.9
Age (years)		
14 to 44	102	52.6
45 to 59	60	30.9
60 or older	32	16.5
Comorbidities		
Diabetes mellitus (n = 163)		
Yes	15	9.2
No	147	97.6
Arterial hypertension (n = 165)		
Yes	61	37.0
No	132	63.0
Coronary disease (n = 165)		
Yes	4	2.4
No	161	97.6
Cerebrovascular disease (n = 165)		
Yes	4	2.4
No	161	97.6
Overweight/obesity (n = 160)		
Yes	100	62.5
No	60	37.5
Visual acuity disorder (n = 163)		
Yes	25	15.2
No	138	84.1
Visual acuity disorder, right eye (n = 192)		
Normal vision	87	44.8
Mild loss	56	28.9
Moderate/severe loss	49	25.3
Visual acuity disorder, right eye		
Normal vision	89	45.9
Mild loss	56	28.9
Moderate/severe loss	49	25.3
Occupational variables		
Average monthly income (CLMMS)		
Below 1	141	72.4
From 1 to 2	46	24.0
More than 2	5	2.6
Daily working hours (n = 138)		
2 to 8	30	21.7
9 to 12	94	68.1
> 12	14	10.1
Well-illuminated workplace (n = 192)		
Yes	186	95.9
No	6	3.1
Illumination with a lamp		
Yes	155	79.9
No	39	20.1
Visual symptoms		
Reduced visual acuity		
Yes	84	43.3
No	110	56.7
Blurred vision in the last month		
Yes	118	60.8
No	75	38.9
Double vision in the last month		
Yes	5	3.0
No	161	97.0
Vision was completely blurred		
Yes	2	1.2
No	164	98.8
Seeing holes or black spots		
Yes	12	7.2
No	154	92.8
Lacrimation in the last month		
Yes	13	7.8
No	153	92.2
Headache in the last 6 months		
Yes	31	18.8
No	134	81.2

CLMMS = current legal minimum monthly salary.

### COMORBIDITIES

The comorbidities identified presented the following prevalences: diabetes mellitus (9.2%), arterial hypertension (37%), coronary disease (24%), cerebrovascular disease (2.4%), overweight or obesity (64.5%) (Table 1).

In relation to visual acuity, it affected 15.2% of workers. When classifying the degree of loss of visual acuity into normal vision, mild loss, and moderate/severe loss, it was observed that 44.8% and 45.9% of workers had normal vision in the right and left eye, respectively, whereas 54.2% of participants presented some type of loss of visual acuity, with 28.9% of cases of mild loss in both right and left eyes, and the other 25.3% of moderate/severe loss of visual acuity (Table 1).

Furthermore, 43.3% of workers had perceived a reduction in visual acuity, and 60.8% reported presenting blurred vision in the last month. Additionally, 18.8% reported episodes of headache in the last 6 months, 7.8% reported lacrimation in the last month, and 7.2% reported seeing holes or black spots in the last month (Table 1).

### SOCIODEMOGRAPHIC AND WORKING CONDITIONS ASSOCIATED WITH LOSS OF VISUAL ACUITY

Statistically significant associations (p < 0.05) were identified between loss of visual acuity with workers’ biological sex and age. The prevalence of loss of visual acuity was 65% higher in men than in women (PR = 1.65, 95% confidence interval [CI] 1.21-2.25). It was also observed that the higher the age, the higher the prevalence of loss of visual acuity. This loss was 4.2 times higher (PR = 5.12, 95%CI 1.10-23.71) for workers aged from 45 to 59 years compared to younger workers, and 20.07 times higher for those aged 60 years or older (PR 21.07, 95%CI 5.15-86.17) (Table 2).

**Table 2:** Sociodemographic and working conditions associated with loss of visual acuity among marketplace workers in 2017 (n = 163)

Characteristic or condition	Loss of visual acuity n (%)		Total n (%)	Chi-square (p-value)^*^	PR (95%CI)
	Yes	No			
Sociodemographic conditions					
Marital status					
With a partner	12 (20.69)	46 (79.31)	58 (35.58)	1.99 (0.158)	1.44 (0.90-2.31)
Without a partner	13 (12.38)	92 (87.62)	105 (64.42)		1.00
Sex					
Men	18 (23.08)	60 (76.92)	78 (47.85)	6.90 (0.008)	1.65 (1.21-2.25)
Women	7 (8.24)	78 (91.76)	85 (52.15)		1.00
Worker’s age (years)					
14 to 44	2 (2.53)	77 (97.47)	79 (48.47)	43.30 (0.000)	1.00
45 to 59	7 (12.96)	47 (87.04)	54 (33.13)		5.12 (1.10-23.71)
60 or older	16 (53.33)	14 (46.67)	30 (18.40)		21.07 (5.15-86.17)
Working conditions					
Daily working hours					
2 to 8	12 (33.33)	24 (66.67)	36 (22.09)	11.48 (0.003)	1.00
9 to 12	11 (10.48)	94 (89.52)	105 (64.42)		0.31 (0.15-0.65)
More than 12	2 (9.09)	20 (90.91)	22 (13.50)		0.27 (0.07-1.10)
Type of illumination - lamp					
Yes	16 (12.31)	114 (87.69)	130 (79.75)		1.00
No	9 (27.27)	24 (63.64)	33 (20.25)	4.54 (0.033)	0.77 (0.57-1.04)

CI = confidence interval; PR = prevalence ratio.

* p < 0.05 = statistically significant associations.

**Figure 1:**
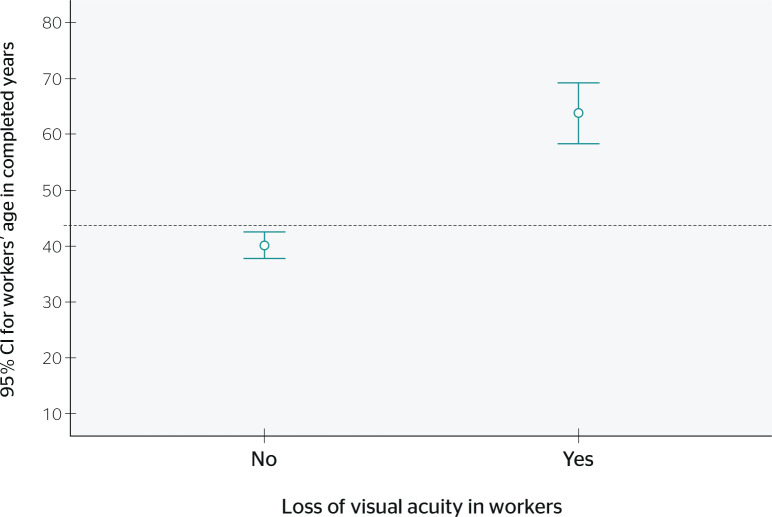
Loss of visual acuity according to workers’ age at a marketplace in a municipality of Southwestern Antioquia, Colombia, 2017 (n = 194). CI = confidence interval.

Statistically significant differences were also identified (t = 7.42, p = 0.000) in mean age among workers with and without loss of visual acuity. Mean age for those with this condition was 63.80 years (± 12.91), a value 23.63 years (95%CI 17.37-29.89) higher than that of workers without loss of visual acuity, whose mean age was 40.17 years (± 15.07 years) (Figure 1).

Despite lack of statistically significant association, a 44% higher prevalence of loss of visual acuity (PR = 1.44) was found in workers with a partner (Table 2).

Conversely, working conditions were significantly associated (p < 0.05) with lower prevalence of loss of visual acuity, since individuals who worked from 9 to 12 hours a day showed a 69% lower prevalence of loss of visual acuity (PR = 0.61, 95%CI 0.15-0.65) than those who worked from 2 and 8 hours a day, and those who did not illuminate their workplace with a lamp had a 33% (PR = 0.67) lower prevalence of loss of visual acuity than those who used this type of illumination in their workplaces (PR = 0.77, 95%CI 0.57-1.04) (Table 2).

### VISUAL SYMPTOMS AND COMORBIDITIES ASSOCIATED WITH LOSS OF VISUAL ACUITY

Statistically significant associations (p < 0.05) were observed between higher prevalence of loss of visual acuity and perceived reduction of visual acuity (PR = 2.08, 95%CI 1.45-2.99) and blurred vision (PR = 2.74, 95%CI 1.39-5.39). Despite lacking statistical significance, the prevalence of loss of visual acuity was higher in workers who reported eye dryness during the month prior to data collection (PR = 1.66), and 81% lower in patients who reported headache in the 6 months prior to data collection (PR = 0.19) (Table 3).

**Table 3:** Visual symptoms and comorbidities associated with loss of visual acuity in marketplace workers for 2017 (n = 163)

Characteristic or condition	Loss of visual acuity n (%)		Total n (%)	Chi-square (p-value)^*^	PR (95%CI)
	Yes	No			
Visual symptoms					
Reduced visual acuity					
Yes	17 (27.42)	45 (72.58)	62 (38.04)	11.24 (0.000)	2.08 (1.45-2.99)
No	8 (7.92)	93 (92.08)	101 (61.96)		1.0
Blurred vision (n = 162)					
Yes	9 (33.33)	18 (66.67)	27 (16.56)	7.96 (0.004)	2.74 (1.39-5.39)
No	16 (11.85)	119 (88.15)	135 (82.82)		1.0
Seeing holes or black spots					
Yes	2 (16.67)	10 (83.33)	12 (7.36)	0.08 (0.776)	1.10 (0.26-4.74)
No	23 (15.23)	128 (84.77)	151 (92.64)		1.0
Eye dryness in the last month					
Yes	3 (23.08)	10 (76.92)	13 (7.98)	0.16 (0.684)	1.66 (0.49-5.60)
No	22 (14.67)	128 (85.33)	150 (92.02)		1.0
Headache in the last 6 months (n = 162)					
Yes	1 (3.33)	29 (96.67)	30 (18.52)	3.07 (0.079)	0.19 (0.03-1.32)
No	24 (18.18)	108 (81.82)	132 (81.48)		1.00
Comorbidities					
Diabetes mellitus (n = 162)					
Yes	3 (20.00)	12 (80.00)	15 (9.26)	10.02 (0.001)	0.25 (0.08-0.74)
No	22 (18.18)	125 (85.03)	147 (90.74)		1.00
Arterial hypertension					
Yes	10 (29.41)	24 (70.59)	34 (20.99)	6.44 (0.011)	2.28 (1.25-4.17)
No	15 (11.72)	113 (88.28)	128 (79.01)		1.00
History of drug use					
Yes	8 (16.67)	40 (83.33)	48 (29.81)	0.07 (0.794)	1.09 (0.58-2.04)
No	17 (15.04)	96 (84.96)	113 (70.19)		1.00

CI = confidence interval; PR = prevalence ratio.

* p < 0.05 = statistically significant associations.

The prevalence of loss of visual acuity was significantly higher (p < 0.05) in workers with arterial hypertension (PR = 2.28, 95%CI 1.25-4.17). Finally, it called the attention that workers with diabetes mellitus had a lower (p < 0.05) prevalence of loss of visual acuity (PR = 0.25, 95%CI 0.08-0.74) (Table 3).

### SOCIODEMOGRAPHIC AND WORKING CONDITIONS, VISUAL SYMPTOMS, AND COMORBIDITIES THAT EXPLAIN LOSS OF VISUAL ACUITY IN WORKERS

#### SOCIODEMOGRAPHIC AND WORKING CONDITIONS

When loss of visual acuity was adjusted for the sociodemographic and occupational variables that were significantly associated with loss of visual acuity or that met the HL goodness-of-fit criteria (p < 0.25), age was found to be the only variable that significantly explains (p < 0.25) a higher prevalence of loss of visual acuity, meaning that the higher the age, the higher the prevalence of this condition, since it was 3.95 times higher among workers aged from 45 to 59 years than among younger workers (adjusted prevalence ratio [PR_a_] = 4.95, 95%CI 2.06-11.88), and 29.81 times higher among workers aged 60 years or older (PR_a_ = 30.81, 95%CI 7.29-129.83) (Table 4).

**Table 4:** Sociodemographic and working conditions, comorbidities, and symptoms that explain loss of visual acuity in workers

Condition - Characteristic	PR^c^	95%CI		PR^a^	95%CI	
		LT	UT		LT	UT
Biological sex						
Men	1.65	1.21	2.25	0.62	0.27	1.43
Age - Category of comparison: 14 to 44 years						
45-59	5.12	1.10	23.71	4.95	2.06	11.88
≥ 60	21.07	5.15	86.17	30.81	7.29	129.83
Marital status						
With a partner	1.44	0.90	2.31	1.56	0.73	3.34
Daily working hours - Category of comparison: ≤ 8 hours						
9 to 12	0.31	0.15	0.65	1.36	0.33	5.58
More than 12	0.27	0.07	1.10	0.87	0.21	3.66
Illuminating the workplace with a lamp						
No	0.77	0.57	1.04	1.50	0.71	3.18

CI = confidence interval; LT = lower threshold; PR_a_ = adjusted prevalence ratio; PR_c_ = crude prevalence ratio; UT = upper threshold.

Despite lacking statistical significance, having a partner (PR_a_ = 1.56), not illuminating the workplace with a lamp (PR_a_= 1.50), and working from 9 to 12 hours a day (PR_a_ = 1.36) also explained higher prevalence of loss of visual acuity. These last two characteristics turned into factors associated with lower prevalence of loss of visual acuity and explained higher prevalence of this condition when adjusted for the other variables included in the analysis (Table 4).

A change was observed with regard to the association between biological sex and prevalence of loss of visual acuity, in which there was an association between higher prevalence of this loss and male sex in crude analysis, but, when sex was adjusted for the other variables, it not only lost its statistical significance but there was also a reversal in the direction of association, thus explaining lower prevalence of loss of visual acuity (Table 4).

#### VISUAL SYMPTOMS AND COMORBIDITIES

When loss of visual acuity was adjusted for the variables that met HL goodness-of-fit criteria and for age, only age and perceived reduction of visual acuity explained this loss significantly (p < 0.05). Loss of visual acuity was greater among workers aged from 45 to 59 years (PR_a_ = 8.55, 95%CI 3.63-20.17) and among those aged 60 years or older (PR_a_ = 64.85, 95%CI 9.60-126.51), maintaining statistical significance and direction of association observed in bivariate analysis. Conversely, perceived reduction of visual acuity explained loss prevalence of loss of visual acuity and maintained statistical significance, but there was a reverse in the direction of association (Table 5).

**Table 5:** Comorbidities and visual symptoms that provide an explanation to loss of visual acuity in workers

Condition - Characteristic	PR^c^	95%CI		PR^a^	95%CI		
		LT	UT		LT	UT	
Age - Category of comparison: 14 to 44 years							
45-49	5.12	1.10	23.71	8.55	3.63	20.17	
≥ 60	21.07	5.15	86.17	34.85	9.60	126.51	
Reduced visual acuity in the last year							
Yes	2.08	1.45	2.99	0.46	0.20	0.98	
Blurred vision in the last month							
Yes	2.74	1.39	5.39	0.56	0.25	1.26	
Headache in the last 6 months							
Yes	0.19	0.03	1.32	5.35	0.98	29.18	
Personal history of diabetes							
Yes	0.25	0.08	0.74	2.55	0.86	7.54	
Personal history of hypertension							
Yes	2.28	1.25	4.17	1.38	0.59	3.25	

CI = confidence interval; LT = lower threshold; PR_a_ = adjusted prevalence ratio; PR_c_ = crude prevalence ratio; UT = upper threshold.

Despite lacking statistical significance, headache (PR_a_ = 5.35), personal history of diabetes (PR_a_ = 2.55), and personal history of hypertension (PR_a_ = 1.38) contributed to explain higher prevalence of loss of visual acuity when adjusted for the other comorbidities and for visual symptoms. Conversely, personal history of hypertension lost its statistical significance and there was a reduction in the strength of association (Table 5).

## DISCUSSION

In 1999, by means of the global initiative “Vision 2020: the right to sight,” the WHO aimed to prevent the development of blindness due to potentially avoidable causes for 2020,^12^ an strategy implemented by Colombia in 2006 through Resolution 4045.^13^ However, despite national and international efforts, visual disability (low vision and blindness) remains a reality, because approximately 2.2 billion people have some type of visual impairment^1^ and, for Colombia, it accounts for 43.2% of all disabilities.^14^ In the present study, 15.2% of the workers were found to have some visual acuity disorder.

One of the relevant risk factors for the analysis of loss of visual acuity is age, which has been related with diseases such as macular degeneration, a condition accounting for approximately 46% of cases of severe loss of visual acuity (20/200-20/400) in individuals older than 40 years.^15^ However, a study in Segovia, Spain, showed that more than 60% of the study population over 40 years of age had a refractive problem,^16^ similar to the findings observed in the present study, in which there was a significant relationship (p < 0.05) between higher age and loss of visual acuity, showing that the higher the age, the stronger the association between age and loss of visual acuity.

Macular degeneration is an entity that leads to severe loss of visual acuity and even blindness,^17^ and one of its risk factors identified in previous studies is smoking, since, as reported by Khan et al.,^17^ individuals who smoke more than 40 packets/year had 2.75 times more risk for loss of visual acuity (*odds ratio* [OR] = 2.75, 95%CI 1.22-6.20). These findings could not be compared with those of the present study, because not all aspects leading to neovascular degeneration or atrophy were assessed. However, it was found that workers with a history of drug use were 9% more likely to present with loss of visual acuity, and further studies with this type of workers are expected to explore the conditions that cause macular degeneration and atrophy in a more detailed manner.

When not using a lamp in the workplace was adjusted with age, marital status, and daily working hours, although this characteristic did not present a significant association, it explained a higher prevalence of loss of visual acuity (PR_a_ = 1.50). However, this condition is difficult to compare, since studies on the topic are still scarce, especially on this type of factors. Only one study conducted in northern Taiwan in a manufacturing company found that loss of visual acuity was higher in night-shift workers (PR = 2.6, 95%CI 1.5-4.4).^18^

Depending on natural lighting and ventilation conditions, the workplace need to be illuminated with a lamp in day and night shifts, what could have happened with the workers included in the present study, who could be further assessed in more detailed studies, in order to provide evidence that can be used to formulate and implement public health strategies in the population with informal jobs in market places.

For the present study, values of visual acuity established by the WHO were re-categorized to identify workers at risk for progression to loss of visual acuity, and a variable named “mild loss of visual acuity” was created to identify workers with vision worse than 20/20 (1. without loss of visual acuity) and better than 20/70 (2. without loss of visual acuity), acknowledging that this category lacks international endorsement and does not classify loss of visual acuity as such. However, for this study, this categorization of the variable should be used to divide workers into workers who were at risk for loss of visual acuity and those who did not, finding that 28.9% of them had mild loss of visual acuity, and those who should be impacted by health promotion and disease prevention actions to prevent the progressive deterioration of visual acuity.

It is important to conduct action aimed at health promotion and eye disease prevention, because people with loss of visual acuity demand greater health care and higher health costs, as shown in a study conducted in the United States which estimated that the annual health care cost of people with macular degeneration is approximately US$ 600.^17^ In Australia, Wang et al.^19^ reported that people with visual acuity deterioration had 3.1 times as likely to use support services provided by the municipality (OR = 3.1. 95%CI 1.8-5.1)^19^; however, these analyses did not separate the population according to their job.

Although the prevalence of loss of visual acuity was initially 75% lower (p < 0.05) in workers with diabetes, when this disease was adjusted for the other comorbidities and for visual symptoms, diabetes started to explain higher prevalence of loss of visual acuity (PR_a_ = 2.55), consistent with the global literature, which reports that diabetes may be considered a vascular retinal disease and that, in developed countries, it is the leading cause of blindness before the age of 60 years, and the third leading cause after this age. Furthermore, it is the most frequent microvascular complication in patients with diabetes, and its incidence also increases with age. The risk for blindness is 25 times higher in patients with diabetes compared to the rest of the population, especially in those with type 1 diabetes,^20^ and reduced visual acuity in diabetic retinopathy is mainly explained by diabetic macular edema, which also causes metamorphopsia and myodesopsias.^21^

Other comorbidity associated with reduced visual acuity is arterial hypertension, and the results of this study, in addition to showing a high prevalence of this disease (20.6%), reveal a significant association (p < 0.05) with visual acuity disorders. Hypertension is the second leading cause of vascular retinal disease, after diabetic retinopathy, which has a prevalence from 7 to 11% in hypertensive patients. Vascular retinal disease is mainly associated with severity, duration, and time of onset of hypertension; furthermore, it often causes reduced visual acuity with symptoms ranging from blurred vision to severe manifestations. If retinal disease is acute, abnormalities disappear as soon blood pressure values are normalized. If it is chronic, there is often no reduced visual acuity, although it may be impaired through indirect mechanisms.^20^ No obstante, lo anterior, the present study did not explore in detail the previously exposed aspects, which limits the comparison of its results with those reported in the literature.

Although since 1996 it has been described that ocular disorders, both organic and functional (defects of refraction or of accommodation), may cause headache through different mechanism, a condition known as secondary headache,^22^ or primary headache, which is the causative agent of eye pain,^23^ this was not a significant factor in the present study. Nonetheless, current literature on loss of visual acuity associated with headache is more related to secondary, acute, emerging causes, such as optic neuritis or temporal arteritis, and even less related with these in a population working in the informal sector of economy or with subsistence jobs.

It is important to bear in mind that, CIs were very wide, albeit significant, for the variable age, and very wide and not significant for the variables headache, diabetes, and hypertension. One of the reasons that explain these results may be the heterogeneity of categories of the compared groups, which is why caution should be taken when interpreting the results, because a type I error may have occurred in the case of age, although there is literature supporting the evidence.
